# Achieving Excellence in the Practice of Chronic Disease Epidemiology

**DOI:** 10.5888/pcd15.180526

**Published:** 2018-11-29

**Authors:** Renee M. Calanan, Michelle Sandoval-Rosario, Janae D. Price, Claudine M. Samanic, Hua Lu, Kamil E. Barbour

**Affiliations:** 1 Centers for Disease Control and Prevention, Atlanta, Georgia; 2 Commissioned Corps, US Public Health Service, Rockville, Maryland; 3 Colorado Department of Public Health and Environment, Denver, Colorado; 4 Arizona Department of Health Services, Phoenix, Arizona; 5 Illinois Department of Public Health, Springfield, Illinois; 6 Indiana State Department of Health, Indianapolis, Indiana

Heart disease, diabetes, cancer, arthritis, and other chronic diseases are the leading causes of death and disability and the leading drivers of health care costs in the United States (1). Health disparities and inequalities exist across chronic diseases, behavioral risk factors, environmental exposures, social determinants, and health care access by sex, race and ethnicity, income, education, disability status, and other social characteristics (2). A white paper developed by the Council of State and Territorial Epidemiologists’ (CSTE’s) Chronic Disease Epidemiology Capacity Building Workgroup stated that for 3 of the Essential Public Health Services — surveillance, communication, and consultation — chronic disease epidemiologists (CDEs) perform functions that are critical to health departments (3). Collecting, analyzing, interpreting, and disseminating data on chronic diseases and related risk factors is vital to understanding and raising awareness about morbidity, mortality, associated costs, and disparities. These data are also vital inputs throughout the process of implementing evidence-based public health approaches to reduce the burden of chronic diseases in the United States.

Chronic disease surveillance is changing, with new priorities that are more upstream, more clinical, more cross-cutting, and more granular than previous priorities; new data sources, such as electronic health records, to supplement traditional sources; and new technologies. Today’s state, territorial, local, and tribal CDEs increasingly need to be strategic, innovative, collaborative, and efficient while wearing many hats and taking on leadership roles: statistician, informaticist, demographer, cartographer, evaluator, communications specialist, privacy officer, strategist, convener, and others. CDEs need to expand partnerships across multiple sectors to leverage data and resources to address social, environmental, and economic conditions that affect health and advance health equity. Timely and locally relevant data, metrics, and analytics are of utmost importance in this work to guide, focus, and assess the effect of prevention initiatives, including those targeting the social determinants of health and enhancing equity (4). Concurrently, chronic disease surveillance is challenged by data gaps, limitations in data access and timeliness, increases in data collection costs, decreases in funding, and inadequate staffing. The CSTE’s *2017 Epidemiology Capacity Assessment Report* enumerated 304 CDEs in all 50 states and the District of Columbia (5). Survey respondents from the 51 jurisdictions indicated a need for 137 additional CDEs (a 45% increase) to reach full capacity, and most (88%) jurisdictions indicated a need to improve capacity in the Essential Public Health Services in chronic disease epidemiology (5).

The public health structure varies across states, and many state public health agencies provide epidemiological technical assistance and resources to local public health agencies. The size, resources, and other demands of local public health agencies might prohibit the hiring of dedicated CDEs or even the ability to have general epidemiologists perform chronic disease epidemiology and surveillance services. In 2016, the National Association of County and City Health Officials conducted a study on the funding, workforce, programs, and partnerships at local public health agencies; 1,930 local public agencies responded to the study survey (6). The survey showed that 49% of local public health agencies directly provided chronic disease epidemiology and surveillance services in the past year; this percentage ranged from 44% to 65% according to the size of the population served: 44% for small populations (<50,000), 56% for medium populations (50,000–499,999), and 65% for large populations (≥500,000) (6). Increasing the number of CDEs to build capacity and enhance expertise in surveillance, communication, and consultation is critically important. The Centers for Disease Control and Prevention’s (CDC’s) State Chronic Disease Epidemiology Assignee Program aims to address the workforce shortage of CDEs in states.

## CDC’s State Chronic Disease Epidemiology Assignee Program

Since 1991, the CDC’s State Chronic Disease Epidemiology Assignee Program has helped states build chronic disease epidemiology capacity by placing a CDC employee (hereinafter referred to as field assignee) in a state or local public health agency. Field assignees assist states by providing epidemiologic consultation and leadership for surveillance systems; offering expertise in designing epidemiological studies, analyzing data, evaluating chronic disease prevention and health promotion programs, and disseminating findings; providing data and identifying priority populations for public health program planning; and mentoring and training entry-level and mid-level CDEs and other staff members in epidemiologic methods and data interpretation.

To date, CDC’s State Chronic Disease Epidemiology Assignee Program has benefited 36 states and New York City during its 28-year history (Figure). Field assignees have served in their state position for up to 12 years. Currently, the program has 4 field assignees; they are in Arizona, Colorado, Illinois, and Indiana. The field assignees’ work has directly enhanced chronic disease epidemiology capacity, and CDC has provided forums and training (eg, introduction to CDC surveillance systems, Evaluation 101, geospatial data methods, Behavior Risk Factor Surveillance System weighting methodology, public health law, legal epidemiology) for the field assignees and state CDEs. Field assignees serve as a liaison between the state or local public health agency and CDC. As a CDC employee, field assignees have access to CDC subject matter experts, training, data sets, analytic software, and an electronic library for broad access to the scientific literature, which can help supplement state resources and further contribute to statewide capacity in the practice of chronic disease epidemiology.

**Figure:**
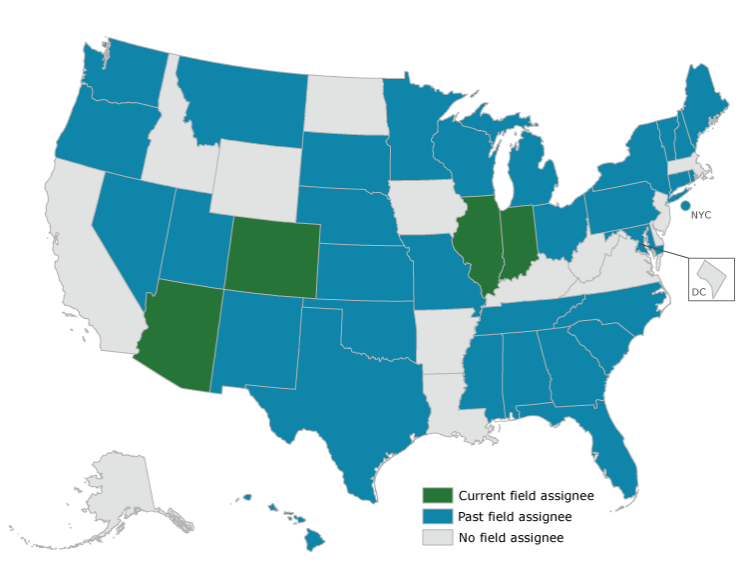
States that have hosted an assignee through the Centers for Disease Control and Prevention’s State Chronic Disease Epidemiology Assignee Program, 1991–2018. The program has benefited 36 states and New York City.

**Table:** 

State or City	Assignee History
Alabama	Past assignee
Alaska	No assignee
Arizona	Current assignee
Arkansas	No assignee
California	No assignee
Colorado	Current assignee
Connecticut	Past assignee
Delaware	No assignee
District of Columbia	No assignee
Florida	Past assignee
Georgia	Past assignee
Hawaii	Past assignee
Idaho	No assignee
Illinois	Current assignee
Indiana	Current assignee
Iowa	No assignee
Kansas	Past assignee
Kentucky	No assignee
Louisiana	No assignee
Maine	Past assignee
Maryland	Past assignee
Massachusetts	No assignee
Michigan	Past assignee
Minnesota	Past assignee
Mississippi	Past assignee
Missouri	Past assignee
Montana	Past assignee
Nebraska	Past assignee
Nevada	Past assignee
New Hampshire	Past assignee
New Jersey	No assignee
New Mexico	Past assignee
New York	Past assignee
New York City	Past assignee
North Carolina	Past assignee
North Dakota	No assignee
Ohio	Past assignee
Oklahoma	Past assignee
Oregon	Past assignee
Pennsylvania	Past assignee
Rhode Island	Past assignee
South Carolina	Past assignee
South Dakota	Past assignee
Tennessee	Past assignee
Texas	Past assignee
Utah	Past assignee
Vermont	Past assignee
Virginia	No assignee
Washington	Past assignee
West Virginia	No assignee
Wisconsin	Past assignee
Wyoming	No assignee

## Accomplishments of Chronic Disease Epidemiology Field Assignees

Field assignees have contributed to capacity building in their states in numerous ways (Box). In recent years, field assignees have focused on analyzing and disseminating state and local data on health disparities and improving data-informed decision-making processes to target public health interventions for chronic disease prevention and management. Colorado’s field assignee has worked to enhance data usage for chronic disease program planning. This field assignee collaborated with a state chronic disease grant program to develop a new data-driven approach to scoring grant applications. This new approach was designed to increase the effect of grantee programs on health disparities by elevating scores of applications proposing to serve areas of greater need. To develop the new approach, a county ranking was created by using a principal components analysis of county data on the burden of disease and the social determinants of health, and a new methodology was developed to apply the results of the county rankings to the scores of grant applicants. Arizona’s field assignee contributed to several state reports to inform program priorities, including the *Arizona American Indian Health Status Summary Report for Data Year 2015 *(7), which was shared statewide with tribal leaders and partners working with tribal communities. The report informed targeted interventions and focused on health disparities. Indiana’s field assignee contributed to several quality improvement initiatives for chronic disease programming and surveillance, such as the development and implementation of out-of-hospital and telemedicine programs for heart disease and heart failure patients in rural areas that lacked both primary care providers and specialists. Illinois’s field assignee applied a novel approach for the state to better understand implementation of evidence-based interventions for high blood pressure and glycemic control among Federally Qualified Health Centers, organizations that serve approximately 1.2 million of Illinois’s most vulnerable citizens. The field assignee is also leading efforts to assess feasibility of a statewide quality improvement collaborative.

**Box. Examples of Responsibilities and Expectations of Assignees in the Centers for Disease Control and Prevention’s State Chronic Disease Epidemiology Assignee Program**

• Provide general epidemiological consultation and assistance to the state public health agency, local public health agencies, and partners as appropriate.

• Ensure collaboration across chronic disease programs and with internal and external stakeholders for epidemiology, surveillance, and evaluation activities.

• Consult with chronic disease program managers about how data can be used to support and target chronic disease prevention efforts and develop strategies for strengthening those efforts.

• Enhance data collection, analysis, interpretation, and dissemination.

• Mentor, develop resources for, and conduct trainings for state and local chronic disease program staff members and epidemiologists to strengthen epidemiology capacity and enhance data usage.

• Serve as preceptor and mentor for student interns, fellows, and preventive medicine residents.

• Build partnerships with other agencies and stakeholders across multiple sectors to increase data sharing and usage.

• Develop and implement chronic disease surveillance plans.

• Develop and implement chronic disease program evaluation plans.

• Contribute to the development of chronic disease and related state plans.

• Provide technical assistance in writing chronic disease-related grant applications, cooperative agreements, and requests for proposals.

• Make presentations at national and local conferences and meetings on behalf of the state public health agency.

• Publish state reports and articles in peer-reviewed scientific journals.

• Participate in Council of State and Territorial Epidemiologists subcommittees and workgroups.

## Past, Present, and Future Capacity-Building Efforts

Many other chronic disease epidemiology capacity-building efforts have occurred or are ongoing. Formal state and local capacity-building programs have included, but are not limited to, CDC/CSTE’s Applied Epidemiology Fellowship and CDC/National Association of Chronic Disease Directors’ Applied Chronic Disease Epidemiology Mentoring Program (8,9). These have been successful programs, but expanded efforts are needed. Governmental agencies, foundations, universities, and others committed to chronic disease–related public health capacity building should collaborate with those working in other subject areas to build capacity on cross-cutting competencies. Examples of topics include those identified by CSTE’s chronic disease epidemiology capacity assessment: using informatics tools in support of epidemiologic practice; understanding institutional review board processes; using systems thinking in epidemiologic planning and policy development; leading community public health planning processes; practicing culturally sensitive epidemiologic activities; conducting program evaluations; and others (10).

Future efforts should build on past and current efforts, be informed by national assessment results, and target jurisdictions with subpar levels of chronic disease epidemiology capacity. Training efforts should be tailored to address changes occurring in public health and chronic disease surveillance. To achieve excellence in chronic disease epidemiology and to build capacity, the following are needed: 1) identify champions for enhancing capacity, 2) continually review and update the essential roles of CDEs, 3) expand the skills and competencies of the current and future workforce, 4) develop and enhance partnerships to improve data sharing, 5) leverage and link existing data sources, 6) improve the availability of local data, 7) fill data gaps to better measure determinants of health and health disparities, and 8) make data more actionable. Strong commitment is vital to building and maintaining capacity-building efforts in chronic disease epidemiology and surveillance in state, territorial, local, and tribal public health agencies. Throughout these capacity-building efforts and across all chronic disease epidemiology and surveillance efforts, the default view must be through a health equity lens.
